# Failure to replicate a robust Down syndrome advantage for maternal well‐being

**DOI:** 10.1111/jir.12808

**Published:** 2021-01-06

**Authors:** M. Jess, S. Flynn, T. Bailey, R. P. Hastings, V. Totsika

**Affiliations:** ^1^ CEDAR University of Warwick Coventry UK; ^2^ Centre for Developmental Psychiatry and Psychology, Department of Psychiatry, School of Clinical Sciences at Monash Health Monash University Melbourne Victoria Australia; ^3^ Division of Psychiatry, Faculty of Brain Sciences University College London London UK

**Keywords:** Down syndrome, Down syndrome advantage, intellectual disability, mothers, psychological well‐being

## Abstract

**Background:**

Family members caring for children with intellectual disability (ID) routinely report heightened levels of psychological distress. However, families of children with Down syndrome typically report better outcomes (known as the Down syndrome advantage). We examined whether the Down syndrome advantage would be present for maternal psychological distress, impact of caregiving, life satisfaction and perceived positive impact of the child with ID when controlling for external variables.

**Methods:**

Mothers of children with Down syndrome (*n* = 111) and mothers of children with ID of mixed aetiologies (*n* = 196) completed measures about their own mental health, perceived impact of caregiving, life satisfaction and perceived positive impact of their child on themselves and the family unit.

**Results:**

A series of group comparisons revealed small to moderate differences supporting the presence of a putative Down syndrome advantage in relation to personal maternal well‐being outcomes. However, when child‐related characteristics and external variables were controlled, the Down syndrome advantage was no longer present, with reduced, small effect sizes observed for all maternal outcomes.

**Conclusions:**

Initial group differences in psychological distress and life satisfaction were largely associated with family poverty, indicating that the Down syndrome advantage may be less robust than previously thought. Future research should seek to move beyond examining the existence of the putative Down syndrome advantage and focus on how families of children with Down syndrome experience family life, including longitudinal research exploring responses to life cycle and transition challenges.

Mothers of children with intellectual disability (ID) experience higher levels of stress, anxiety and more symptoms of depression than mothers of children without disabilities (Baker *et al*. 2002; Eisenhower *et al*. 2005; Hayes and Watson 2013; Totsika *et al*. 2011a). Increased levels of maternal psychological distress have an early onset (during the preschool years) and persist into adulthood (Orsmond *et al*. 2003; Benson and Kersh 2011; Estes *et al*. 2013). There is, however, variation in the experience of psychological distress in mothers related to their child's genetic condition (Adams *et al*. 2018). In particular, existing research suggests that mothers of children with Down syndrome have better psychological outcomes than mothers of children with other conditions associated with ID (e.g. cerebral palsy and autism) (Hodapp *et al*. 2003; Abbeduto *et al*. 2004; Blacher and McIntyre 2006; Pisula 2007; Griffith *et al*. 2010; Blacher *et al*. 2013). This phenomenon is commonly referred to as the ‘Down syndrome advantage’ and has been evidenced in mothers across their child's lifespan (Dabrowska and Pisula 2010).

The Down syndrome advantage has predominantly been evidenced by lower levels of psychological problems in mothers. More recently, researchers have examined the positive effects of raising a child with a disability (Hastings and Taunt 2002; Ricci and Hodapp 2003; Corrice and Glidden 2009; Skotko *et al*. 2011; Hastings 2016). Children with Down syndrome are often described as being sociable, cheerful (Walz and Benson 2002) and affectionate (Wishart and Johnston 1990). The presence of these characteristics may influence parents to be more affectionate, increase the amount of positive interactions and hold more positive perceptions about their child. Indeed, mothers of children with Down syndrome have reported that they are better rewarded by and have closer relationships with their child compared with mothers of children with other developmental disabilities (e.g. autism and fragile‐X syndrome) (Hodapp *et al*. 2001; Abbeduto *et al*. 2004). In general, however, the Down syndrome advantage has been explored less often in relation to parents' positive outcomes.

More recently, there has been debate as to whether the Down syndrome advantage is truly a robust diagnostic group difference or whether it is driven by factors distinctly separate from, or associated with, the syndrome itself. Other characteristics of the child may be one source for the observed group differences. One such factor is behaviour problems, as these have long been associated with heightened maternal stress, anxiety and depression in families of children with ID (Hastings 2002; Hodapp *et al*. 2003; Johnston *et al*. 2003; Ricci and Hodapp 2003; Tomanik *et al*. 2004; Estes *et al*. 2009). Parents of children with Down syndrome report lower levels of child behaviour problems in comparison with other children with ID (Hodapp *et al*. 2003; Blacher and McIntyre 2006). In addition to fewer behaviour problems, children with Down syndrome often have comparatively high levels of prosocial and adaptive behaviours which may, in turn, be associated with better maternal mental health outcomes (Beck *et al*. 2004; Blacher and McIntyre 2006; Neece and Baker 2008; Totsika *et al*. 2015).

In addition to the characteristics of the child, factors external to the child may also explain the Down syndrome advantage findings. Mothers of children with Down syndrome are more likely to be older (Loane *et al*. 2013), and older maternal age is often associated with better psychological and family adjustment in mothers of children with and without disabilities (Benzies *et al*. 2013; Mayberry *et al*. 2007; Trute *et al*. 2012). Furthermore, Stoneman (2007) reported that household income was significantly higher for families raising children with Down syndrome than in families raising other children with ID. These results have also been found elsewhere in the literature, although these differences were not statistically significant (Eisenhower *et al*. 2005; Corrice and Glidden 2009; Glidden *et al*. 2014). Stoneman found that mothers of children with Down syndrome reported significantly lower levels of stress and depressive symptoms, but that this group difference was not evident when the variance attributable to family income was controlled. It is possible, therefore, that the perceived Down syndrome advantage may be attributable to older maternal age and/or reduced family poverty.

Despite the abundance of research demonstrating better mental health and more positive outcomes for parents of children with Down syndrome, it is not possible to draw firm conclusions about the putative Down syndrome advantage. The group difference is sometimes robust to controlling for other key variables and sometimes not. For example, Corrice and Glidden (2009) reported a Down syndrome advantage in maternal well‐being when compared with mothers of other children with ID. However, when maternal age and child adaptive behaviours were controlled, group differences were no longer apparent. In contrast, Eisenhower *et al*. (2005) found that mothers of preschool children with Down syndrome reported lower levels of stress and depression than mothers of children with cerebral palsy or autism. When differences in behaviour problems were accounted for, the child's diagnostic group still significantly contributed to maternal stress.

Contradictory findings emphasise the importance of accounting for covariates when examining the existence of a Down syndrome advantage. The aim of the current study was to determine whether the Down syndrome advantage would be present in both personal (psychological distress and life satisfaction) and child‐related (impact of caregiving and positive gains) maternal outcomes when multiple child and maternal variables (e.g. child age, child behavioural and emotional problems, and family poverty) were carefully controlled. Specifically, the outcomes of mothers of children with Down syndrome were compared with those of mothers of other children with ID. In contrast to much of the existing literature (Abbeduto *et al*. 2004; Griffith *et al*. 2010), mothers of children with autism were excluded from the comparison group in this study as this design is vulnerable to inflating the presence of an advantage. This potential biasing of results by comparison with an autism group may be due to higher rates of problem behaviours (Griffith *et al*. 2010; Totsika *et al*. 2011a) and limitations in prosocial skills and communication (Griffith *et al*. 2010) in children with autism as well as increased psychological distress in parents (Griffith *et al*. 2010). Based on previous research, we expect to find a putative Down syndrome advantage in this sample. Further, we expect that any apparent Down syndrome advantage would be explained by controlling for child or family factors.

## Method

### Participants

Three hundred and seven biological, adoptive or foster mothers of children with ID were included in this study in either the Down syndrome or mixed aetiology ID group. Children with Down syndrome were not excluded if they also had a diagnosis of autism spectrum disorder (ASD) as reported by the survey respondent. However, children in the comparison group were excluded if they had ASD. Overall, children in this study were aged between 4 and 15 years (*M* = 8.85 years, *SD* = 3.01). Table 1 outlines participant demographic data by participant group. Participants in this study were a subsample of a larger dataset of participants who took part in the telephone survey component of the 1000 Families Study (Hastings *et al*. 2020), a large cohort study of families with a child with ID in the UK (*N* = 1184). The 1000 Families Study did not include a question about participants' (i.e. mothers') age, due to a request from a research ethics committee to reduce to a minimum the collection of personal data about family carers. Thus, we are unable to include maternal age in the present study, although this covariate is potentially important in explaining the putative Down syndrome advantage.

**TABLE 1:** Participant demographics

	Down syndrome (*n* = 111)	Mixed ID (*n* = 196)
Relationship to child		
Biological mother	107 (96.4%)	173 (88.3%)
Adoptive mother	2 (1.8%)	20 (10.2%)
Foster mother	2 (1.8%)	3 (1.5%)
Education level		
No qualifications	–	2 (1.0%)
Some GCSEs	5 (4.5%)	16 (8.2%)
5 + GCSEs	1 (0.9%)	9 (4.6%)
A/AS levels	7 (6.3%)	14 (7.1%)
Higher education	26 (23.4%)	48 (24.5%)
Degree	65 (58.6%)	97 (49.5%)
Don't know	–	2(1.0%)
Employment		
Do something else	12 (10.8%)	20 (10.2%)
In a job and currently working	49 (44.1%)	63 (32.1%)
On maternity/paternity leave	1 (0.9%)	5 (2.6%)
Self‐employed	8 (7.2%)	25 (12.8%)
Doing voluntary work	4 (3.6%)	5 (2.6%)
Looking after home and family	33 (29.7%)	75 (38.3%)
Unemployed	3 (2.7%)	2 (1.0%)
Married or living with partner	93 (83.8%)	154 (80.2%)
Median weekly household income	Between £600 and £700	Between £500 and £600
Median IMD decile	6	6
Not managing financially	8 (7.2%)	26 (13.3%)
Struggle to raise £2000 in an emergency	35 (31.5%)	82 (42.7%)
Mean family poverty composite (SD)	0.97 (SD = 0.99)	1.30 (SD = 1.11)
Mean child age in years (range; SD)	8.35 (4–15; SD = 2.93)	9.15 (4–15; SD = 3.02)
Child sex		
Male	66 (59.5%)	115 (58.7%)
Female	45 (40.5%)	79 (40.3%)
Mean DBC (range; SD)	55.58 (1–125; SD = 26.30)	65.65 (13–142; SD = 29.26)
Mean VABS communication score (range; SD)	64.75 (31–108; SD = 13.03)	60.33 (25–104; SD = 14.90)
Mean VABS socialisation score (range; SD)	66.22 (27–101; SD = 12.93)	59.51 (31–104; SD = 13.02)

DBC, Developmental Behaviour Checklist; GCSE, General Certificate of Secondary Education; ID, intellectual disability; IMD, Index of Multiple Deprivation; VABS, Vineland Adaptive Behaviour Scale.

### Measures

#### Positive Gains Scale

The Positive Gains Scale (Jess *et al*. 2020; Pit‐ten Cate 2003) was used to measure parental perceptions of the positive impact of their child. Five items reflect the perceived benefits of raising a child (e.g. ‘since having this child I have grown as a person’), and two reflect positive gains for the family (e.g. ‘since having this child, my family has become closer to one another’). Lower scores indicate greater positive gain. This measure has good reliability for mothers of children with developmental disabilities (Jess *et al*. 2020). Cronbach's alpha in the current study was 0.84 for the Down syndrome group and 0.77 for the ID comparison group.

#### Kessler 6

The Kessler 6 (Kessler *et al*. 2002) is a six‐item self‐report measure developed to screen for the presence of psychological distress in non‐clinical community samples. Participants were asked to score each item using a five‐point Likert scale [0 (*symptom not at all present*) to 4 (*symptom present over time*)] about their own psychological distress over the past 30 days. Higher scores indicate greater levels of distress. The Kessler 6 maintains excellent psychometric properties in mothers of children with ID (Totsika *et al*. 2011b). Cronbach's alpha in the current study was 0.84 for both the Down syndrome group and the ID group.

#### Impact of caregiving

A seven‐item ‘Impact of caregiving on carer’ scale from the Survey of Informal Carers in Households 2009/10 (NHS Digital 2010) was used. Participants were asked to indicate whether certain aspects of their lives have been affected by caring for their child. These were unable to socialise or take part in social or leisure activities at all (due to caring responsibilities), reduced time with spouse or partner, reduced time with other family members, reduced time with friends, difficulties making new friends, reduced time spend doing sport or physical activity and reduced time spent doing a pastime or hobby. Participants were asked to select any of seven leisure activities (all that applied) that had been negatively affected by the care they provide to their child with ID. A higher summed number of options chosen on this scale indicate a higher negative impact of caregiving on participants. Kuder–Richardson 20 for the Down syndrome group (0.75) and the ID group (0.75) showed adequate internal consistency of the measure.

#### Life satisfaction

A single‐item measure asked participants to rate their general life satisfaction on a scale of 1 (*completely dissatisfied*) to 10 (*completely satisfied*). This scale has been used within major UK social surveys with large‐normative samples (*n* = 1000+), including the Millennium Cohort Study (Plewis 2007). Single‐item life satisfaction measures have been found to correlate strongly with other, long‐form, well‐established life satisfaction measures (e.g. Cheung and Lucas 2014).

#### Family poverty

Family poverty was measured using a composite variable created by the research team incorporating four single‐item indicators of poverty:

1. Total weekly household income was dichotomised into above or below the UK median weekly household income (£677.83) at the time of the study; with those categorised as earning above the UK median income scoring 0 and those earning below it scoring 1.
2. How participants felt that they were financially managing was dichotomised with those who were living comfortably, doing alright or just about getting by scoring 0 and those who were finding it quite or very difficult scoring 1.
3. How likely it would be that participants could raise £2000 in 1 week was dichotomised such that those who could easily raise the money or raise it with some minimal sacrifices scored 0 and those who would have to do something drastic or could not raise the £2000 in 1 week scored 1.
4. Participants in the bottom quintile of neighbourhoods on the Index of Multiple Deprivation were considered to be living in deprived environments and scored 1, all others scored 0. Index of Multiple Deprivation scores were gathered from family residential postcodes, using scores (which combine a number of indices of social deprivation for the area to form a relative ranking) from the Office for National Statistics (2019).

These four dichotomised indicators were summed to create the family poverty composite (for all cases when participants had responded to at least three of the four items), such that scores ranged from 0 to 4 and that higher scores indicated greater levels of poverty. Notably, these indicators were measured at the family level, as compared with maternal‐level education and employment, which were, accordingly, included separately to the composite.

#### Vineland Adaptive Behaviour Scale

The Vineland Adaptive Behaviour Scale II – Survey form (VABS II; Sparrow *et al*. 2005) was used to measure child adaptive behaviour. This semistructured interview measure contains a range of items that provide an assessment of adaptive behaviour across four domains: socialisation, communication, daily living skills and motor skills (the motor skills domain is used for children under 7 years old only). The items in each domain are arranged in developmental order, and not all questions are asked in an interview, instead the interviewer estimates an adaptive level and asks in detail about skill items in this range to arrive at an accurate estimate of a child's abilities. Each item is scored using a three‐point scale [0 (*Never or almost never*), 1 (*Sometimes*), 2 (*Usually*)]. The socialisation and communication domain standard scores (representing skills potentially enhanced in Down syndrome) were used in the present analysis.

#### Developmental Behaviour Checklist

The Developmental Behaviour Checklist – Parent (DBC‐P; Einfeld and Tonge 1992a) is a 96‐item measure of behavioural and emotional problems in children and adolescents with ID. Each item is scored on a three‐point Likert scale [0 (*not as far as you know*) to 2 (*very often or true*)]. For the current study, the total behaviour problem score of all items was used as an overall measure of emotional and behavioural problems. The DBC‐P has good reliability in studies of children with ID (Einfeld and Tonge 1992a), and the Cronbach's alpha for the current study was 0.93 for the Down syndrome group and 0.94 for the ID group.

### Procedure

Ethical approval was granted by the NHS Health Research Authority NRES Committee West Midlands – South Birmingham Research Ethics Committee (15/WM/0267). Study participants were recruited through special schools, social media advertising and advertising through disability charities. Study packs were distributed directly to parents (e.g. via the child's school) and included an information sheet, consent form, the survey questionnaire and a prepaid return envelope. Participants could request a study pack online by following a link on social media. Participants were also able to complete the survey online. Informed consent was obtained, either on paper or online (depending on survey completion method), before participants completed the survey. Within the survey, participants were asked whether they would be willing to take part in a telephone interview, and those who consented were contacted by a researcher to complete the VABS and DBC‐P in the context of a semistructured interview over the telephone.

Of the 1184 participants in the total 1000 Families Study sample, 985 were (adoptive, biological or foster) mothers, and 599 of these had completed the VABS and DBC‐P. Of these 599 participants, 111 reported that their child had Down syndrome. The remaining 488 participants were identified as having a child with ID other than Down syndrome, of which 292 had a parent‐reported co‐diagnosis of autism (and thus were excluded from the current study). The remaining subsample of 307 participants (196 in the mixed aetiology ID group and 111 in the Down syndrome group) is that analysed within the present study.

### Analysis

Analyses involved the comparison of the two groups: Mothers who had a child with Down syndrome and mothers who had a child with ID in the mixed aetiology group. Independent sample *t* tests were used to compare the mean scores of the four maternal well‐being outcome variables between the two groups. For the final set of models, analyses of covariance were run for each of the four outcomes, this time including all five covariates in each analysis of covariance to examine if any Down syndrome advantage was robust to controlling for family and child variables. In preliminary checks, we examined correlations to check for multicollinearity between the predictor variables. No evidence of multicollinearity was found.

Cohen's *d* was used to estimate the effect size of potential mean differences between the two groups. Cohen's *d* was estimated by calculating the mean difference between the two study groups, and then dividing the result by the pooled standard deviation. Confidence intervals (CIs) for effect sizes were also calculated.

Given that there were a number of children with Down syndrome for whom parents reported the presence of autism (*n* = 14), it is possible that a putative Down syndrome advantage would have been slightly attenuated using our design approach. Therefore, we also ran sensitivity analyses in which these families were excluded from the comparisons and report the results of these briefly in addition to the primary analyses.

## Results

### Unadjusted group differences

Maternal outcomes (psychological distress, life satisfaction, positive gain and impact of caregiving) were compared between the two groups using *t* tests to test for the presence of a putative Down syndrome advantage. Mean scores for each group and Cohen's *d* effect sizes for the differences are summarised in Table 2. A statistically significant difference was present for both personal maternal psychological outcomes. Mothers of children with Down syndrome reported less psychological distress: Cohen's *d* = 0.42, 95% CI [0.18, 0.65] and greater life satisfaction: Cohen's *d* = 0.30, 95% CI [0.06, 0.53], than mothers of other children with ID; which represent medium‐sized effects. However, neither child‐related maternal outcome (positive gains or impact of caregiving) differed statistically between mothers of children with Down syndrome and the other ID groups, and the associated effect sizes were very small.

**TABLE 2:** Unadjusted means for maternal outcomes by study group

Maternal outcomes	Down syndrome		Other IDs		*t* (*df*)	Effect size (*d*)	95% CI for effect size	
	Mean	SD	Mean	SD	Down syndrome vs. other ID		LL	UL
Psychological distress	6.67	4.62	8.70	4.99	3.13* (220)	0.42	0.18	0.65
Life satisfaction	6.81	1.92	6.23	1.98	−2.103* (220)	0.30	0.06	0.53
Positive gains	29.42	4.24	28.74	3.82	−1.484 (220)	0.16	−0.06	0.40
Impact of caregiving	4.23	1.74	4.61	1.80	−0.138 (205)	0.22	−0.03	0.46

CI, confidence interval; ID, intellectual disability; LL, lower level; UL, upper level.

* *P* < 0.05.

When the children with Down syndrome who were reported as having autism were removed from the study group, the pattern of results remained unchanged except that the (negative) impact of caring was then also found to be significantly lower in the Down syndrome group (*P* = 0.016; *d* = 0.25, CI [0.04, 0.47]) but still with a small effect size.

### Adjusted group differences

After controlling for child behavioural and emotional problems (DBC‐P), child communication and socialisation skills, family poverty and child age (as an approximation for maternal age, which was not measured), significant group differences were no longer present for the personal maternal well‐being outcomes ( Table 3). Specifically, there was no longer a statistically significant main effect of the group, and the associated effect sizes were very small: for psychological distress (*F*_1,272_ = 3.21, *P* = 0.07), Cohen's *d* = 0.20 and life satisfaction (*F*_1,272_ = 1.88, *P* = 0.172), Cohen's *d* = 0.15. Family poverty (*F*_1,272_ = 22.81, *P* < 0.001) and maternal unemployment (*F*_1,272_ = 105.62, *P* = 0.02) were associated with higher levels of psychological distress. Further, family poverty (*F*_1,272_ = 12.14, *P* = 0.001) and maternal unemployment (*F*_1,272_ = 16.49, *P* < 0.001) were also associated with lower levels of life satisfaction.

**TABLE 3:** Analysis of covariance summary for maternal well‐being outcomes

Covariates	Psychological distress			Life satisfaction			Positive gains			Impact of caregiving		
	*F*	*P*	*d*	*F*	*P*	*d*	*F*	*P*	*d*	*F*	*P*	*d*
Child age	6.285	0.577		0.294	0.588		0.226	0.635		0.531	0.467	
DBC total	67.248	0.069		0.376	0.540		3.929	0.049		0.268	0.605	
Communication	5.385	0.606		0.400	0.528		2.262	0.134		0.001	0.978	
Socialisation	3.910	0.660		0.022	0.881		0.237	0.627		1.637	0.202	
Family poverty	22.813	<0.001		0.018	0.893		1.368	0.243		12.138	0.001	
No degree	7.685	0.538		0.296	0.587		1.668	0.198		0.008	0.929	
No job	105.621	0.023		0.705	0.402		0.957	0.329		16.490	<0.001	
Study group	64.831	0.074	0.197	1.011	0.316	0.121	0.689	0.407	0.101	1.880	0.172	0.152

DBC, Developmental Behaviour Checklist.

Again, as shown in Table 3, there were no study group differences and very small effect sizes for positive gains (*F*_1,272_ = 1.01, *P* = 0.32), Cohen's *d* = 0.20 and impact of caregiving (*F*_1,253_ = 0.69, *P* = 0.41), Cohen's *d* = 0.15. The DBC total score was found to be a predictor for gains (*F*_1,253_ = 3.93, *P* = 0.049).

When the children with Down syndrome who were reported as having autism were removed from the study group in the adjusted analyses, the Down syndrome group differences remained unchanged except for psychological distress. In the reduced model, psychological distress was marginally lower in the Down syndrome group (*P* = 0.049) but still with a small effect size (*d* = 0.26).

## Discussion

Unadjusted comparisons provided support for the existence of a Down syndrome advantage for personal maternal outcomes (reduced psychological distress and increased life satisfaction), although not for maternal outcomes related to their child (impact of caregiving and positive gains). Removing those with autism also from the Down syndrome group also resulted in a group difference for impact of caregiving. When controlling for child and family characteristics, this apparent Down syndrome advantage was not present for either maternal psychological distress or life satisfaction. Further, family poverty and maternal unemployment were significantly associated with both increased psychological distress and decreased life satisfaction. These findings support previous research that suggests that a Down syndrome advantage may not be robust to controlling for other important differences between families of children with Down syndrome and other families (Stoneman 2007; Corrice and Glidden 2009). In the current study, the main factor associated with the initial Down syndrome advantage was lower levels of poverty in the families of children with Down syndrome, replicating other research findings (Stoneman 2007).

Child factors in the current study did not appear to explain the Down syndrome advantage (other than the anticipated impact of autism – which was examined in sensitivity analyses and had a small impact on the findings). Although child behavioural and emotional problems scores were lower for the Down syndrome group, it was not significantly associated with any outcomes. Similarly, no associations were found between maternal outcomes and adaptive behaviour scores, in contrast to previous research (Beck *et al*. 2004; Blacher and McIntyre 2006; Neece and Baker 2008; Totsika *et al*. 2015), although children with Down syndrome had higher communication and socialisation skills (Table 1).

We did not have data on maternal age, and this is a key limitation of this study. Future research would benefit from including maternal age as a covariate to determine whether older maternal age and relative family socio‐economic advantage are related. In the current study, the comparator group included ID of mixed aetiologies as opposed to one specific ID diagnosis. Arguably, covariates included in this study may have different patterns of association with outcome measures in specific ID aetiological groups. For example, differences in the communication and socialisation domains of the VABS (Sparrow *et al*. 2005) have been found between five genetic syndromes (Prader–Willi, fragile‐X, Williams, Down and Angelman) (Di Nuovo and Buono 2011) and children with Prader–Willi or Williams syndrome have more behaviour problems, as rated on the DBC (Einfeld and Tonge 1992b), than children with Down or fragile‐X syndrome (Einfeld *et al*. 1999). It is important that, if these differential interactions do exist, they are identified as they will have implications for clinical practice.

It is important to point out that we did find a Down syndrome advantage: mothers of children with Down syndrome reported lower levels of psychological distress and better quality of life. We have reported evidence that this ‘advantage’ may be driven primarily by better socio‐economic circumstances in families of children with Down syndrome. Future research should examine this association in more detail, especially to identify what is different in the experiences of families of children with Down syndrome that is related to their relatively improved socio‐economic status. Research needs to move beyond repeated examination of whether a Down syndrome advantage exists to explaining what happens in the lives of parents and families. This may include longitudinal research to understand how families of children with Down syndrome experience and respond to life cycle and transition challenges and also qualitative research to understand parents' perceptions and experiences and approach to family life.

## Source of funding

This study was funded by Cerebra and the Economic and Social Research Council Doctoral Training Centre at the University of Warwick.

## Conflict of interest

The authors declare no conflict of interest.

## Data availability statement

No data are available. Data from this study are not available for sharing due to ethical approval requirements. Researchers interested in collaboration should contact the corresponding author with their expression of interest.
